# Mechanical knowledge, but not manipulation knowledge, might support action prediction

**DOI:** 10.3389/fnhum.2014.00737

**Published:** 2014-09-17

**Authors:** François Osiurak

**Affiliations:** ^1^Laboratoire d'Etude des Mécanismes Cognitifs (EA 3082), Université de LyonBron Cedex, France; ^2^Institut Universitaire de France, Maison des UniversitésParis, France

**Keywords:** affordance, apraxia, manipulation knowledge, mechanical knowledge, tool use

Bach et al. (2014) proposed a novel model of action understanding, the affordance-matching hypothesis, to explain how people both interpret and predict actions of others. This model is based on two types of information. The first is function knowledge and is supposed to inform people about the goals that can be achieved with tools. The second is manipulation knowledge and is thought to provide information about the motor behaviors required to achieve these goals. In their model, function knowledge and manipulation support action interpretation and action prediction, respectively. Here, I mainly discuss the idea that manipulation knowledge might be central to action prediction.

The distinction made by Bach et al. (2014) between function knowledge and manipulation knowledge is inspired to some extent from a part of the literature on apraxia (e.g., Buxbaum and Saffran, 2002; van Elk et al., 2014). In their model, function knowledge is viewed as storing information about the goals of tools, namely, their usual function^1^. For instance, as they wrote, people know that “a tap is for getting water.” By contrast, manipulation knowledge would be useful to determine what are the motor behaviors required to use tools (e.g., knowing that a tap requires turning it clockwise). This way of conceptualizing the cognitive bases of human tool use has however been intensively debated in recent years. Particularly, a growing body of evidence indicates a strong link in left brain-damaged apraxic patients between the ability to actually use familiar tools (i.e., the use of a tool with its corresponding object, such as a hammer with a nail) and the ability to use novel tools to solve mechanical problems (Goldenberg and Hagmann, 1998; Goldenberg and Spatt, 2009; see also Osiurak et al., 2009; Jarry et al., 2013; Osiurak et al., 2013). In line with this, it has been proposed that mechanical knowledge, but not manipulation knowledge, might be central to tool use, by allowing people to reason about physical object properties (Osiurak et al., 2010, 2011; Goldenberg, 2013; Osiurak, 2014). Contrary to Bach et al. (2014), the mechanical knowledge hypothesis posits that what people learn when using a tap is not that a clockwise rotation of the hand is needed, but rather that a clockwise rotation of the tap is needed. In this framework, motor behaviors are adjusted on-line on the basis of the prediction of the tool use action to be done. Interestingly, a strong link between mechanical knowledge and the left inferior parietal lobe has also been documented, challenging the role of this cerebral region for the storage of manipulation knowledge (for reviews, see Goldenberg, 2013; Orban and Caruana, 2014; Osiurak, 2014).

Another important aspect concerns the role of function knowledge. Patients with a selective impairment of function knowledge have been shown to be still able to actually use familiar tools with their corresponding objects as well as to use novel tools to solve mechanical problems (for a review, see Osiurak et al., 2011). In other words, function knowledge is neither sufficient nor necessary for tool use (Buxbaum et al., 1997). So the intriguing issue is, what is the role of function knowledge? It has been recently proposed that function knowledge might be useful for determining the social usages associated with tools (Osiurak et al., 2010, 2011; see also Goldenberg, 2013; Osiurak, 2014). For example, function knowledge can help someone to know that a knife can be used to cut tomatoes or meat, open an envelope, peel a fruit, and so on. However, this knowledge is not viewed as supporting tool use *per se*. After all, people can know that a stethoscope can be found in a medical context and that its function is to “listen to the heart” without being able to use it properly. To do so, mechanical knowledge is required. Consequently, as Bach et al. (2014) suggested, function knowledge can indeed be of primary interest to interpret the actions of others, by determining in function of the context and of the social usages associated with the tool the potential goals of the action.

Having said this, I propose to revise their model by modifying the idea that action prediction is supported by manipulation knowledge (see Figure 1). Rather, I assume that people might predict the outcomes of the actions made by others by using mechanical knowledge. To illustrate it, let us come back to an example given by Bach et al. (2014). As they stated: “Imagine, for example, the unpleasant situation of standing across from another person holding a gun. Object knowledge specifies that a gun is for shooting (function knowledge), and that, in order to achieve this goal, the gun would have to be raised, pointed at the target, and fired (manipulation knowledge)” (Bach et al., 2014; p. 3). In this example, Bach et al. (2014) implied that the position of the gun to be correctly used as well as its utilization derive from manipulation knowledge. However, it is also possible to stress that mechanical knowledge is needed to guide the user to correctly position the gun and to use it. In addition, the issue is how manipulation knowledge can help you to know that the bullet can kill you. This is purely independent from the motor behaviors of the user. However, this prediction can vary according to whether you wear bulletproof vest or not. In other words, to know whether the bullet will kill you or not, you need mechanical knowledge to compare the physical properties of the bullet with those of your body or of your bulletproof vest. Again, in this case, manipulation knowledge is absolutely unnecessary to predict the outcomes of the action.

**Figure 1:**
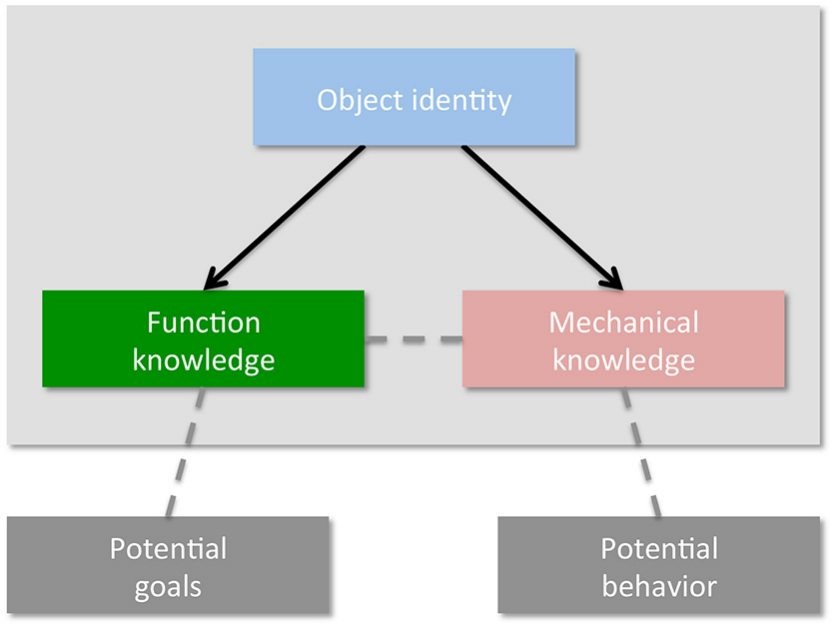
**Revised version of the model of action understanding of Bach et al. (2014)**.

In sum, the model proposed by Bach et al. (2014) provides an appropriate account to think about the potential sources of information at the basis of action interpretation and prediction. However, I am not convinced that manipulation knowledge is the appropriate theoretical construct that can explain how people predict the actions of others. Before concluding, I would like to emphasize that the revised model I propose can be viewed as a strong version of the mechanical knowledge hypothesis, excluding any role for manipulation knowledge in action understanding. This might appear surprising considering the significant literature supporting the importance of this knowledge for action and object representation (for recent publications, see Yee et al., 2013; Buxbaum, 2014; Buxbaum et al., 2014a,b). In a way, there is here an apparent discrepancy raising the key issue of whether the brain stores mechanical and/or manipulation knowledge. The available evidence is not sufficient to answer it, suggesting interesting perspectives for future research in the field.

## Conflict of interest statement

The author declares that the research was conducted in the absence of any commercial or financial relationships that could be construed as a potential conflict of interest.

## Footnotes

^1^Note that, contrary to Bach et al. (2014), many authors even assume that function knowledge is the basis for predicting actions of others because it might contain information about the specific actions associated with the physical properties of tools (e.g., van Elk et al., 2014). Nevertheless, I will not discuss this aspect in more detail here.
